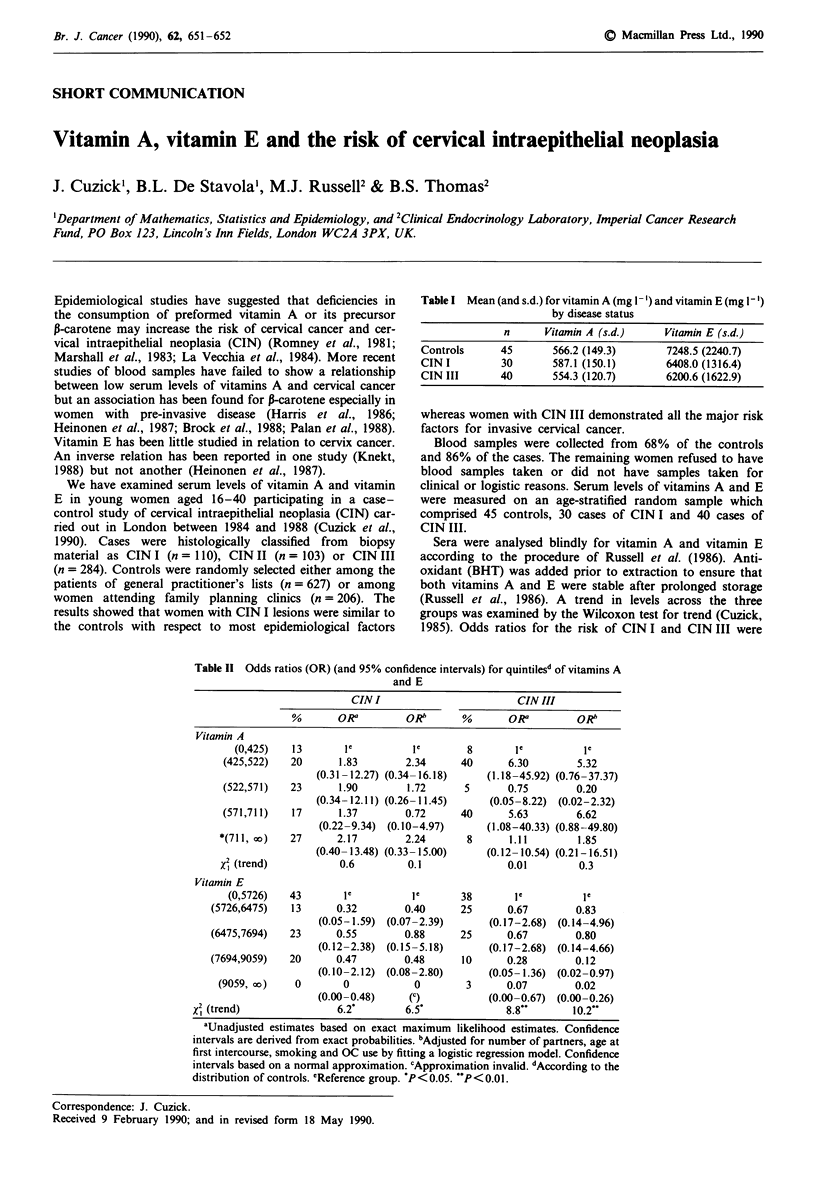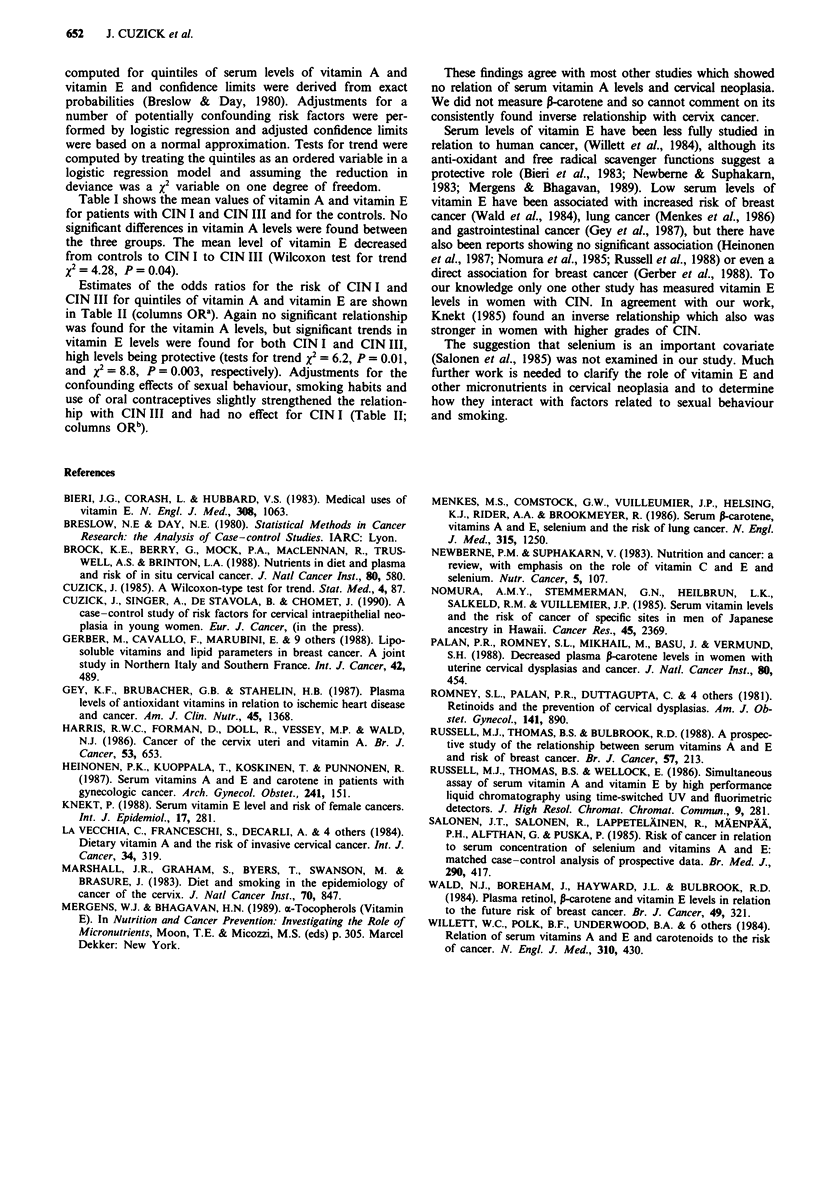# Vitamin A, vitamin E and the risk of cervical intraepithelial neoplasia.

**DOI:** 10.1038/bjc.1990.348

**Published:** 1990-10

**Authors:** J. Cuzick, B. L. De Stavola, M. J. Russell, B. S. Thomas

**Affiliations:** Department of Mathematics, Statistics and Epidemiology, Imperial Cancer Research Fund, Lincoln's Inn Fields, London, UK.


Br. J. Cancer (1990), **62**, 651–652 © Macmillan Press Ltd., 1990

### SHORT COMMUNICATION

### Vitamin A, vitamin E and the risk of cervical intraepithelial neoplasia

J. Cuzick[1], B.L. De Stavola[1], M.J. Russell[2] & B.S. Thomas[2]

[1]*Department of Mathematics, Statistics and Epidemiology, and* [2]*Clinical Endocrinology Laboratory, Imperial Cancer Research Fund, PO Box 123, Lincoln's Inn Fields, London WC2A 3PX, UK.*

Epidemiological studies have suggested that deficiencies in the consumption of preformed vitamin A or its precursor β-carotene may increase the risk of cervical cancer and cervical intraepithelial neoplasia (CIN) (Romney *et al.*, 1981; Marshall *et al.*, 1983; La Vecchia *et al.*, 1984). More recent studies of blood samples have failed to show a relationship between low serum levels of vitamins A and cervical cancer but an association has been found for β-carotene especially in women with pre-invasive disease (Harris *et al.*, 1986; Heinonen *et al.*, 1987; Brock *et al.*, 1988; Palan *et al.*, 1988). Vitamin E has been little studied in relation to cervix cancer. An inverse relation has been reported in one study (Knekt, 1988) but not another (Heinonen *et al.*, 1987).

We have examined serum levels of vitamin A and vitamin E in young women aged 16–40 participating in a case-control study of cervical intraepithelial neoplasia (CIN) carried out in London between 1984 and 1988 (Cuzick *et al.*, 1990). Cases were histologically classified from biopsy material as CIN I ($n = 110$), CIN II ($n = 103$) or CIN III ($n = 284$). Controls were randomly selected either among the patients of general practitioner's lists ($n = 627$) or among women attending family planning clinics ($n = 206$). The results showed that women with CIN I lesions were similar to the controls with respect to most epidemiological factors whereas women with CIN III demonstrated all the major risk factors for invasive cervical cancer.

**Table I** Mean (and s.d.) for vitamin A (mg l$^{-1}$) and vitamin E (mg l$^{-1}$) by disease status

| | *n* | *Vitamin A (s.d.)* | *Vitamin E (s.d.)* |
|---|---|---|---|
| Controls | 45 | 566.2 (149.3) | 7248.5 (2240.7) |
| CIN I | 30 | 587.1 (150.1) | 6408.0 (1316.4) |
| CIN III | 40 | 554.3 (120.7) | 6200.6 (1622.9) |

Blood samples were collected from 68% of the controls and 86% of the cases. The remaining women refused to have blood samples taken or did not have samples taken for clinical or logistic reasons. Serum levels of vitamins A and E were measured on an age-stratified random sample which comprised 45 controls, 30 cases of CIN I and 40 cases of CIN III.

Sera were analysed blindly for vitamin A and vitamin E according to the procedure of Russell *et al.* (1986). Antioxidant (BHT) was added prior to extraction to ensure that both vitamins A and E were stable after prolonged storage (Russell *et al.*, 1986). A trend in levels across the three groups was examined by the Wilcoxon test for trend (Cuzick, 1985). Odds ratios for the risk of CIN I and CIN III were

**Table II** Odds ratios (OR) (and 95% confidence intervals) for quintiles[d] of vitamins A and E

| | *CIN I* | | | *CIN III* | | |
|---|---|---|---|---|---|---|
| | % | *OR*[a] | *OR*[b] | % | *OR*[a] | *OR*[b] |
| *Vitamin A* | | | | | | |
| (0,425) | 13 | 1[e] | 1[e] | 8 | 1[e] | 1[e] |
| (425,522) | 20 | 1.83 | 2.34 | 40 | 6.30 | 5.32 |
| | | (0.31–12.27) | (0.34–16.18) | | (1.18–45.92) | (0.76–37.37) |
| (522,571) | 23 | 1.90 | 1.72 | 5 | 0.75 | 0.20 |
| | | (0.34–12.11) | (0.26–11.45) | | (0.05–8.22) | (0.02–2.32) |
| (571,711) | 17 | 1.37 | 0.72 | 40 | 5.63 | 6.62 |
| | | (0.22–9.34) | (0.10–4.97) | | (1.08–40.33) | (0.88–49.80) |
| *(711, ∞) | 27 | 2.17 | 2.24 | 8 | 1.11 | 1.85 |
| | | (0.40–13.48) | (0.33–15.00) | | (0.12–10.54) | (0.21–16.51) |
| $\chi_1^2$ (trend) | | 0.6 | 0.1 | | 0.01 | 0.3 |
| *Vitamin E* | | | | | | |
| (0,5726) | 43 | 1[e] | 1[e] | 38 | 1[e] | 1[e] |
| (5726,6475) | 13 | 0.32 | 0.40 | 25 | 0.67 | 0.83 |
| | | (0.05–1.59) | (0.07–2.39) | | (0.17–2.68) | (0.14–4.96) |
| (6475,7694) | 23 | 0.55 | 0.88 | 25 | 0.67 | 0.80 |
| | | (0.12–2.38) | (0.15–5.18) | | (0.17–2.68) | (0.14–4.66) |
| (7694,9059) | 20 | 0.47 | 0.48 | 10 | 0.28 | 0.12 |
| | | (0.10–2.12) | (0.08–2.80) | | (0.05–1.36) | (0.02–0.97) |
| (9059, ∞) | 0 | 0 | 0 | 3 | 0.07 | 0.02 |
| | | (0.00–0.48) | ([c]) | | (0.00–0.67) | (0.00–0.26) |
| $\chi_1^2$ (trend) | | 6.2* | 6.5* | | 8.8** | 10.2** |

[a]Unadjusted estimates based on exact maximum likelihood estimates. Confidence intervals are derived from exact probabilities. [b]Adjusted for number of partners, age at first intercourse, smoking and OC use by fitting a logistic regression model. Confidence intervals based on a normal approximation. [c]Approximation invalid. [d]According to the distribution of controls. [e]Reference group. *$P<0.05$. **$P<0.01$.

Correspondence: J. Cuzick.
Received 9 February 1990; and in revised form 18 May 1990.

computed for quintiles of serum levels of vitamin A and vitamin E and confidence limits were derived from exact probabilities (Breslow & Day, 1980). Adjustments for a number of potentially confounding risk factors were performed by logistic regression and adjusted confidence limits were based on a normal approximation. Tests for trend were computed by treating the quintiles as an ordered variable in a logistic regression model and assuming the reduction in deviance was a $\chi^2$ variable on one degree of freedom.

Table I shows the mean values of vitamin A and vitamin E for patients with CIN I and CIN III and for the controls. No significant differences in vitamin A levels were found between the three groups. The mean level of vitamin E decreased from controls to CIN I to CIN III (Wilcoxon test for trend $\chi^2 = 4.28$, $P = 0.04$).

Estimates of the odds ratios for the risk of CIN I and CIN III for quintiles of vitamin A and vitamin E are shown in Table II (columns OR[a]). Again no significant relationship was found for the vitamin A levels, but significant trends in vitamin E levels were found for both CIN I and CIN III, high levels being protective (tests for trend $\chi^2 = 6.2$, $P = 0.01$, and $\chi^2 = 8.8$, $P = 0.003$, respectively). Adjustments for the confounding effects of sexual behaviour, smoking habits and use of oral contraceptives slightly strengthened the relationship with CIN III and had no effect for CIN I (Table II; columns OR[b]).

These findings agree with most other studies which showed no relation of serum vitamin A levels and cervical neoplasia. We did not measure β-carotene and so cannot comment on its consistently found inverse relationship with cervix cancer.

Serum levels of vitamin E have been less fully studied in relation to human cancer, (Willett *et al.*, 1984), although its anti-oxidant and free radical scavenger functions suggest a protective role (Bieri *et al.*, 1983; Newberne & Suphakarn, 1983; Mergens & Bhagavan, 1989). Low serum levels of vitamin E have been associated with increased risk of breast cancer (Wald *et al.*, 1984), lung cancer (Menkes *et al.*, 1986) and gastrointestinal cancer (Gey *et al.*, 1987), but there have also been reports showing no significant association (Heinonen *et al.*, 1987; Nomura *et al.*, 1985; Russell *et al.*, 1988) or even a direct association for breast cancer (Gerber *et al.*, 1988). To our knowledge only one other study has measured vitamin E levels in women with CIN. In agreement with our work, Knekt (1985) found an inverse relationship which also was stronger in women with higher grades of CIN.

The suggestion that selenium is an important covariate (Salonen *et al.*, 1985) was not examined in our study. Much further work is needed to clarify the role of vitamin E and other micronutrients in cervical neoplasia and to determine how they interact with factors related to sexual behaviour and smoking.

### References

BIERI, J.G., CORASH, L. & HUBBARD, V.S. (1983). Medical uses of vitamin E. *N. Engl. J. Med.*, **308**, 1063.

BRESLOW, N.E & DAY, N.E. (1980). *Statistical Methods in Cancer Research: the Analysis of Case-control Studies.* IARC: Lyon.

BROCK, K.E., BERRY, G., MOCK, P.A., MACLENNAN, R., TRUSWELL, A.S. & BRINTON, L.A. (1988). Nutrients in diet and plasma and risk of in situ cervical cancer. *J. Natl Cancer Inst.*, **80**, 580.

CUZICK, J. (1985). A Wilcoxon-type test for trend. *Stat. Med.*, **4**, 87.

CUZICK, J., SINGER, A., DE STAVOLA, B. & CHOMET, J. (1990). A case-control study of risk factors for cervical intraepithelial neoplasia in young women. *Eur. J. Cancer*, (in the press).

GERBER, M., CAVALLO, F., MARUBINI, E. & 9 others (1988). Liposoluble vitamins and lipid parameters in breast cancer. A joint study in Northern Italy and Southern France. *Int. J. Cancer*, **42**, 489.

GEY, K.F., BRUBACHER, G.B. & STAHELIN, H.B. (1987). Plasma levels of antioxidant vitamins in relation to ischemic heart disease and cancer. *Am. J. Clin. Nutr.*, **45**, 1368.

HARRIS, R.W.C., FORMAN, D., DOLL, R., VESSEY, M.P. & WALD, N.J. (1986). Cancer of the cervix uteri and vitamin A. *Br. J. Cancer*, **53**, 653.

HEINONEN, P.K., KUOPPALA, T., KOSKINEN, T. & PUNNONEN, R. (1987). Serum vitamins A and E and carotene in patients with gynecologic cancer. *Arch. Gynecol. Obstet.*, **241**, 151.

KNEKT, P. (1988). Serum vitamin E level and risk of female cancers. *Int. J. Epidemiol.*, **17**, 281.

LA VECCHIA, C., FRANCESCHI, S., DECARLI, A. & 4 others (1984). Dietary vitamin A and the risk of invasive cervical cancer. *Int. J. Cancer*, **34**, 319.

MARSHALL, J.R., GRAHAM, S., BYERS, T., SWANSON, M. & BRASURE, J. (1983). Diet and smoking in the epidemiology of cancer of the cervix. *J. Natl Cancer Inst.*, **70**, 847.

MERGENS, W.J. & BHAGAVAN, H.N. (1989). α-Tocopherols (Vitamin E). In *Nutrition and Cancer Prevention: Investigating the Role of Micronutrients*, Moon, T.E. & Micozzi, M.S. (eds) p. 305. Marcel Dekker: New York.

MENKES, M.S., COMSTOCK, G.W., VUILLEUMIER, J.P., HELSING, K.J., RIDER, A.A. & BROOKMEYER, R. (1986). Serum β-carotene, vitamins A and E, selenium and the risk of lung cancer. *N. Engl. J. Med.*, **315**, 1250.

NEWBERNE, P.M. & SUPHAKARN, V. (1983). Nutrition and cancer: a review, with emphasis on the role of vitamin C and E and selenium. *Nutr. Cancer*, **5**, 107.

NOMURA, A.M.Y., STEMMERMAN, G.N., HEILBRUN, L.K., SALKELD, R.M. & VUILLEMIER, J.P. (1985). Serum vitamin levels and the risk of cancer of specific sites in men of Japanese ancestry in Hawaii. *Cancer Res.*, **45**, 2369.

PALAN, P.R., ROMNEY, S.L., MIKHAIL, M., BASU, J. & VERMUND, S.H. (1988). Decreased plasma β-carotene levels in women with uterine cervical dysplasias and cancer. *J. Natl. Cancer Inst.*, **80**, 454.

ROMNEY, S.L., PALAN, P.R., DUTTAGUPTA, C. & 4 others (1981). Retinoids and the prevention of cervical dysplasias. *Am. J. Obstet. Gynecol.*, **141**, 890.

RUSSELL, M.J., THOMAS, B.S. & BULBROOK, R.D. (1988). A prospective study of the relationship between serum vitamins A and E and risk of breast cancer. *Br. J. Cancer*, **57**, 213.

RUSSELL, M.J., THOMAS, B.S. & WELLOCK, E. (1986). Simultaneous assay of serum vitamin A and vitamin E by high performance liquid chromatography using time-switched UV and fluorimetric detectors. *J. High Resol. Chromat. Chromat. Commun.*, **9**, 281.

SALONEN, J.T., SALONEN, R., LAPPETELÄINEN, R., MÄENPÄÄ, P.H., ALFTHAN, G. & PUSKA, P. (1985). Risk of cancer in relation to serum concentration of selenium and vitamins A and E: matched case-control analysis of prospective data. *Br. Med. J.*, **290**, 417.

WALD, N.J., BOREHAM, J., HAYWARD, J.L. & BULBROOK, R.D. (1984). Plasma retinol, β-carotene and vitamin E levels in relation to the future risk of breast cancer. *Br. J. Cancer*, **49**, 321.

WILLETT, W.C., POLK, B.F., UNDERWOOD, B.A. & 6 others (1984). Relation of serum vitamins A and E and carotenoids to the risk of cancer. *N. Engl. J. Med.*, **310**, 430.